# Susceptibility of the Non-Targeted Crustacean *Eurytemora affinis* to the Endocrine Disruptor Tebufenozide: A Transcriptomic Approach

**DOI:** 10.3390/genes12101484

**Published:** 2021-09-24

**Authors:** Caroline Arcanjo, Gauthier Trémolet, Nathalie Giusti-Petrucciani, Aurélie Duflot, Joëlle Forget-Leray, Céline Boulangé-Lecomte

**Affiliations:** UMR-I 02 Environmental Stresses and Biomonitoring of Aquatic Ecosystems (SEBIO), FR CNRS 3730 SCALE, Université Le Havre Normandie, CEDEX, 76063 Le Havre, France; gauthier.tremolet@univ-lehavre.fr (G.T.); nathalie.giusti@univ-lehavre.fr (N.G.-P.); duflota@univ-lehavre.fr (A.D.); joelle.leray@univ-lehavre.fr (J.F.-L.); celine.lecomte@univ-lehavre.fr (C.B.-L.)

**Keywords:** biomarkers, copepods, insect growth regulators, pesticides, transcriptomics

## Abstract

Copepods are zooplanktonic crustaceans ubiquitously widespread in aquatic systems. Although they are not the target, copepods are exposed to a wide variety of pollutants such as insect growth regulators (IGRs). The aim of this study was to investigate the molecular response of a non-targeted organism, the copepod *Eurytemora affinis*, to an IGR. Adult males and females were exposed to two sub-lethal concentrations of tebufenozide (TEB). Our results indicate a sex-specific response with a higher sensitivity in males, potentially due to a differential activation of stress response pathways. In both sexes, exposure to TEB triggered similar pathways to those found in targeted species by modulating the transcription of early and late ecdysone responsive genes. Among them were genes involved in cuticle metabolism, muscle contraction, neurotransmission, and gametogenesis, whose mis-regulation could lead to moult, locomotor, and reproductive impairments. Furthermore, genes involved in epigenetic processes were found in both sexes, which highlights the potential impact of exposure to TEB on future generations. This work allows identification of (i) potential biomarkers of ecdysone agonists and (ii) further assessment of putative physiological responses to characterize the effects of TEB at higher biological levels. The present study reinforces the suitability of using *E. affinis* as an ecotoxicological model.

## 1. Introduction

Copepods are small crustaceans inhabiting most aquatic environments from freshwater to marine and brackish systems. The Copepoda subclass has 10 orders [1] and 14,724 known species (Encyclopedia Of Life, accessed on 4 March 2021, https://eol.org/), including free-living or parasitic organisms, which shows a high diversity in morphology and physiology [2]. These organisms are important in the trophic chain as they link primary producers with larger predators [3]. They provide essential ecosystem services by supporting the maintenance of fish of economic importance. They are thus suitable model organisms for ecotoxicology and genomics studies [2,4,5] due to their primordial position in the trophic chain, copepod ubiquity, diversity, and capacity to transfer pollutants to higher trophic levels [6,7,8,9,10,11,12].

‘Omics’ technologies (e.g., transcriptomics, proteomics, and metabolomics) and ecotoxicology are utilised in ecotoxicogenomics to assess toxicant impacts, from a mechanistic point of view, on organisms that are representative of ecosystems [13,14]. Use of these ‘omics’ tools after exposure to toxic compounds could help in the detection of early stress responses to contamination and thus could define early molecular biomarkers of exposure in organisms. Recent advances in non-model genome sequencing have allowed the use of these technologies for a greater number of species commonly used in ecotoxicology, such as copepods [14]. 

Among toxic compounds released in ecosystems, pesticides and particularly toxic compounds that behave as endocrine disruptors (EDs) are of growing concern. EDs are defined as ‘exogenous substances or mixtures that possess properties that might be expressed to lead to endocrine disruption in an intact organism, or its progeny, or (sub) populations’ [15]. EDs can (i) alter hormone secretion, (ii) interfere with hormone receptors, or (iii) modify the metabolism of circulating hormones [16]. Insect growth regulators (IGRs) are specifically designed to disrupt ‘processes essential to the normal development of insects or their progeny’ by altering moulting [17]. Despite their selectivity, these molecules could affect the endocrine systems of non-targeted organisms in the vicinity. In insects, moult is controlled by ecdysteroid 20-hydroxyecdysone (20E). It binds to the EcR-USP (ecdysteroid receptor-ultraspiracle) receptor complex, which leads to the transcriptional up-regulation of early response genes. This results in the transcriptional regulation of late-responsive genes involved in moulting processes. In crustaceans, these processes are considered similar; thus, putative moulting pathways were proposed based on insect and decapods studies [18,19,20,21]. Therefore, information on the endocrine systems in copepods was originally derived from knowledge of decapods despite differences between these groups [22]. Furthermore, studies on the effects of EDs on different crustacean groups, including copepods, show diverse responses (ED did not affect all groups) (reviewed in [23]). This highlights the complexity of extrapolation from one group to another. Therefore, in environmental risk assessment, it is essential to evaluate the impact of EDs on non-targeted organisms, such as copepods, that have ecological importance. 

In view of the above, the aim of the present work was to explore the molecular response of a non-targeted organism to insect growth regulators via a transcriptomic approach. The calanoid copepod *E. affinis—*a species with widespread presence in Northern Hemisphere estuaries [24]—was chosen as a model. *E. affinis* is a suitable test organism due to its small size, sexual dimorphism, short generation time, and ease of culturing in the laboratory [5,25]. This species has been used in several ecotoxicological studies to assess toxicant effects by (i) assessing whole-organism responses including survival, development, reproduction, and behaviour [26,27,28,29,30,31,32] and (ii) molecular investigations of gene expression [33,34]. Copepods were exposed to sub-lethal concentrations (0.5 and 50 µg/L), of a model insecticide, tebufenozide (TEB). This IGR behaves as an ecdysone receptor agonist that mimics a 20E moulting hormone. It targets lepidopteran larvae inducing a lethal precocious moult [35,36]. Despite its narrow spectrum against lepidopteran larvae [37], the effects of TEB on non-targeted species such as crustaceans (i.e., with similar moulting systems) remain to be assessed in copepods. 

## 2. Materials and Methods 

### 2.1. Copepod Sampling and Acclimatization

*E. affinis* copepods were sampled at ebb tide in July 2019 in the oligo-mesohaline zone of the Seine estuary at the Tancarville station (latitude 49 28′19.24″ N, longitude 0 27′55.303″ E, Normandie, France). They were collected in a Working Party (WP) 2 plankton net (200 µm mesh size) and immediately washed through four successive sieves to ensure the elimination of large particles and predators. Copepods were collected on 500 µm and 250 µm mesh size sieves and transferred into isotherm containers in Seine water and brought back to the laboratory. Once in the laboratory, salinity was gradually adjusted to 15 psu (practical salinity unit) using a mixture of UV-treated filtered sea water (Deauville, Normandie, France) and distilled water. Copepods were then collected through 250 µm mesh size sieves and transferred to a clean 15 psu water glass tank. They were fed *ad libitum* with *Rhodomonas salina* algae and kept under optimal conditions (i.e., 15 °C, 15 psu, 18-h/6-h light-dark cycle, [38]) for at least three days before exposure.

### 2.2. Chemical Preparation and Experimental Design

Tebufenozide (TEB; CAS Number 112410-23-8; analytical standard) was purchased from Sigma-Aldrich (Germany). Stock solutions at 5 mg/L and 500 mg/L were prepared in acetone (Sigma-Aldrich, Germany)and were extemporaneously diluted in experimental devices containing 15 psu filtered (0.4 µm) water to yield final concentrations of 0.5 µg/L and 50 µg/L. Selection of the sub-lethal concentrations was based on toxicity tests in *E. affinis* (Figure S1) and literature data showing effects of a 0.5 µg/L TEB exposure in *Gammarus fossarum* [39] and a maximum expected concentration of 50 µg/L in water (lake) after TEB application in a nearby forest [40]. The final acetone concentration in all experimental devices was 0.01% *w/v*, in accordance with OECD (Organisation for Economic Cooperation and Development) guidelines [41]. Water quality controls in all experimental devices were performed at T0 and T+72h to maintain the TEB concentration during experiment based on HPLCMS with a quantification limit of 0.4 µg/L (La Drôme Laboratoire, Valence, France).

Prior to the experiment, devices (i.e., glass crystallizers) were saturated at least 48 h with 15 psu filtered contaminated water to avoid contaminant adsorption during further exposure. Exposure was then performed under optimal conditions (i.e., 15 °C, 15 psu, 18-h/6-h light-dark cycle) using depurated copepods distributed in triplicate within the saturated experimental devices containing 400 mL (1 copepod/mL) of fresh contaminated 15 psu filtered water. The solvent control exposure was conducted under the same conditions as the TEB exposure, with a final acetone concentration of 0.01% *w/v.* Exposure solutions were daily renewed (50% of the media). Copepods were fed *ad libitum* with *R. salina* algae every exposure day prior to the solution renewal. After 72 h of exposure, triplicates of 30 males and 30 females (without eggs) were made. Pools were washed with RNA-free water, dried by removing the washing water, quickly frozen in liquid nitrogen, and stored at −80 °C until used for RNA extraction.

### 2.3. RNA Extraction and Library Preparation and Sequencing

RNA extractions were performed using the ZR Tissue & Insect RNA Microprep kit (Zymo Research, Irvine, CA, USA) following the manufacturer’s instructions after crushing and homogenization with a Precellys homogenizer (Bertin Technologies, France). RNA quantity was determined using a QuantiFluor ^®^ RNA kit on a Quantus™ Fluorometer (Promega, France) and qualified on a RNA 6000 Pico RNA chip on a 2100 Bioanalyzer (Agilent Technologies, Les Ulis, France). 

The library preparation and sequencing were performed by Biofidal (Biofidal, Vaulx-en-Velin, France, http://www.biofidal-lab.com accessed on 20 September 2021). Briefly, cDNA libraries were prepared from 200 ng of total RNA using the Universal Plus mRNA-Seq Library Preparation with a NuQuant kit (NuGen Tecan Genomics, Switzerland). cDNA was quantified with a QuantiFluor ^®^ dsDNA kit (Promega, France) and NuQuant^®^ (Nugen Tecan, Switzerland) on a Qubit^®^ 2.0 Fluorometer (Thermo Fisher Scientific, France). The quality was assessed on a High-Sensitivity DNA chip on a 2100 Bioanalyzer (Agilent Technologies, Santa Clara, CA, USA). Libraries were run on a High and Mid output Flow Cell NextSeq 500 instrument (Illumina, San Diego, CA, USA), with a single-end read (75 and 150 base pair (bp)). The sequencing strategy was established based on the aim of the present paper: to determine differential expression. To do so, single-end reads were shown to be sufficient [42]. For the high output run, SR 75 bp, the sequencing was repeated in order to reach the total number of expected reads. The reads produced after sequencing were homogeneous with good quality score (Table S1).

### 2.4. Differential Gene Expression

Data pre-processing, gene count generation, and statistical analysis were performed by Biofidal (Biofidal, Vaulx-en-Velin, France, http://www.biofidal-lab.com accessed on 20 September 2021). Raw reads were first submitted to a quality selection (Phred score above 30) and adapter trimming using Trimmomatic [43]. Reads with length < 36 bp were also removed. Between 7 and 24 million reads per sample were produced after pre-processing. Reads were aligned to the *E. affinis* reference genome (GCA_000591075.2 Eaff_2.0; Atlantic clade, [44]) with the STAR aligner [45], and gene count generation was performed using FeatureCounts [46]. The count matrix was used to perform PCA and to generate a heatmap of the Euclidean distance (Figure S2), showing good clustering between biological replicates. Indeed, the PCA results highlighted that 98% of the variance was explained by a biological variable (i.e., sex) underlying no the batch effect produced by the use of two flow-cells for the sequencing. The observed dispersion between replicates of the same group found in the PCA (Female 50 µg/L replicate 1 and Female control replicate 1) was likely due to biological diversity among replicates more than a batch effect as they were sequenced on different flow-cells. Thus, all sequenced samples were included for the differential analysis. Assessment of differential gene expression was performed using the R packages DESeq2 [47] and EdgeR [48]. Genes with adjusted *p*-value (FDR) ≤ 0.05 and |FoldChange| ≥ 2 were considered significantly differentially expressed. Genes satisfying those thresholds with both methods were considered to be a refined subset of differentially expressed genes (DEGs).

### 2.5. Functional Analysis

To gain insight into the DEG biological functions, gene ontology (GO) enrichment was performed. Prior to the enrichment, a list of *E. affinis* gene identifiers linked to their related GO terms was produced using Blast2GO v.5.2.2 [49] to overcome the lack of such a published database. Briefly, sequences of all genes were aligned against the BLAST non-redundant (nr) protein database using the blastx algorithm at the e-value cutoff of 1 × 10^−3^ with a 30-blast hit. GO annotation of successfully blasted sequences was performed using Blast2GO mapping and annotation tools using default settings. To improve GO annotation, sequences were also search against the InterPro data base running InterProScan and using the annotation expender (ANNEX) within Blast2GO. The GO enrichment analysis was then performed with the R package topGO v.2.36.0 [50] on the DEGs against the GO annotation file produced in Blast2GO using a Fisher’s exact test with the “classic” algorithm. GO terms corresponding to biological process (BP), cellular component (CC) or molecular function (MF) were considered significantly enriched with a Fisher’s exact test *p*-value ≤ 0.01. From 19% to 45% of the DEGs were considered for BP, from 18% to 40% for CC, and from 25% to 61% for MF. To gain an overview of all DEG functions, we manually searched each DEG with known annotation (genome annotation download from NCBI in October 2019). Protein names were search within the UniProt Knowledgebase (UniProtKB, https://www.uniprot.org/ accessed on 20 September 2021) database filtered by organisms (“crustacea” or “arthropoda” as a priority).

To complete the GO enrichment, DEGs as well as all *E. affinis* genes in fasta format were annotated against the KEGG (Kyoto Encyclopedia of Genes and Genomes) database using the KASS (KEGG Automatic Annotation Server) interface. The single directional best hit method was chosen with BLAST as the search program. *Daphnia pulex* and *Penaeus vannamei* were selected organisms as they were the only two crustaceans that were annotated in the database. The files produced, i.e., gene identifier associated with the ko number, were used to retrieve KEGG pathways corresponding to each ko number using the R package KEGGREST v.1.24.1 [51]. The KEGG pathway enrichment analysis was performed using contingency tables with genes in KEGG pathways for all *E. affinis* genes and for DEGs by applying Fisher’s exact test. *P*-values were then corrected (FDR), and pathways with FDR ≤ 0.05 were considered significantly enriched. Volcano as well as dot plots were drawn using the *ggplot2* v3.2.0 R package [52], and Venn diagrams were drawn with the VennDiagram v1.6.20 R package [53]. 

## 3. Results

### 3.1. Tebufenozide Concentration in Water

*E. affinis* copepods were exposed to nominal concentrations of 0.5 and 50 µg/L of TEB. Water was sampled at the beginning (T0) and the end (T72h) of exposure to measure the concentration of TEB. Analysis revealed that the concentration of TEB was below the quantification limit (0.4 µg/L) in the control and in the 0.5 µg/L exposure to TEB at T0 and T72h (Table 1). Concentrations of TEB were 13.82 µg/L at T0 and 11.73 µg/L at T72h for the 50 µg/L nominal concentration exposure, which showed a decrease of approximately 15% in the experiment (Table 1). To avoid confusion with the concentration of TEB in water, nominal concentrations of TEB (i.e., 0.5 and 50 µg/L) will be referred to as nominal in this publication.

### 3.2. Differential Expression Analysis

**Figure 1:**
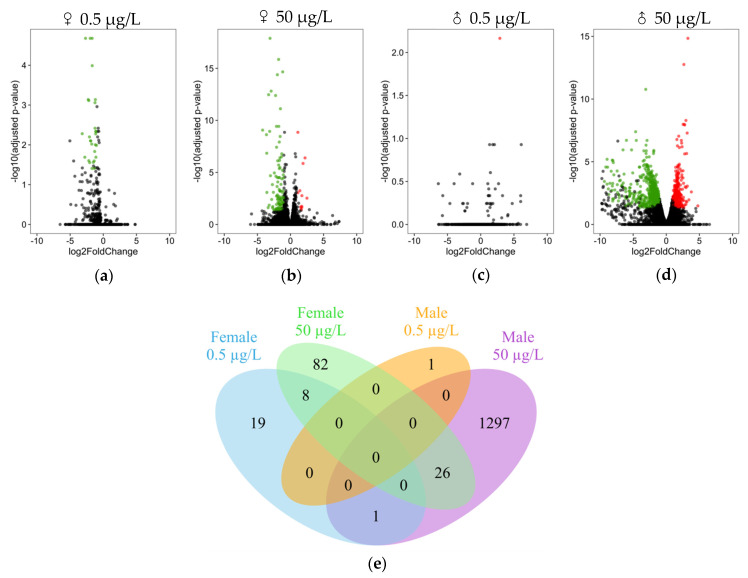
Differentially expressed genes (DEGs) in female (**a**,**b**) and male (**c**,**d**) copepods exposed to 0.5 µg/L (**a**,**c**) and 50 µL/L (**b**,**d**) of TEB. Genes with |FoldChange| ≥ 2 and an adjusted *p*-value ≤ 0.05 were considered mis-regulated. Down-regulated genes are represented in green and up-regulated genes in red. (**e**) Venn diagram of DEGs shared between sexes and TEB concentrations. Green and red dots respective down- and up- regulated genes with their log2FoldChange. If not significant, dots were grey.

Differential expression analysis was performed to determine the effects of a model endocrine disruptor with known modes of action, tebufenozide, on the gene expressions of male and female *E. affinis*. Overall, the number of differentially expressed genes (DEGs; adjusted *p*-value ≤ 0.05 and |FoldChange| ≥ 2) increased with the concentration of TEB for both sexes. In this study, 28 genes in females and one gene in males were mis-regulated after exposure at 0.5 µg/L (Figure 1a,c), whereas 116 and 1324 genes were highlighted in females and males, respectively, at 50 µg/L (Figure 1b,d). Under all conditions, excluding those in which males were exposed to 0.5 µg/L of TEB, genes were mostly down-regulated and represented from 65 to 100% of DEGs. No DEGs were shared in males and females for both concentrations of TEB (Figure 1e). Few genes overlapped between conditions, eight genes overlapped between females for both concentrations of TEB and 26 genes between males and females at 50 µg/L (Figure 1e). A gene coding for *mucin-5AC-like* (LOC111697205) was identified among the female overlapping genes at both concentrations. Furthermore, among the 26 genes shared between sexes for the highest exposure to TEB, we found two genes that code for proteins involved in muscle contraction, i.e., myosins (LOC111711689 and LOC111711716), one gene involved in moulting, i.e., *methyl farnesoate epoxydase-like* (LOC111717477), and two *hsp70* genes (LOC111707610 and LOC111717003). The complete list of DEGs in males and females at concentrations of 0.5 and 50 µg/L of TEB and the list of shared genes are reported in Table S2.

### 3.3. DEG Functional Analysis

**Figure 2:**
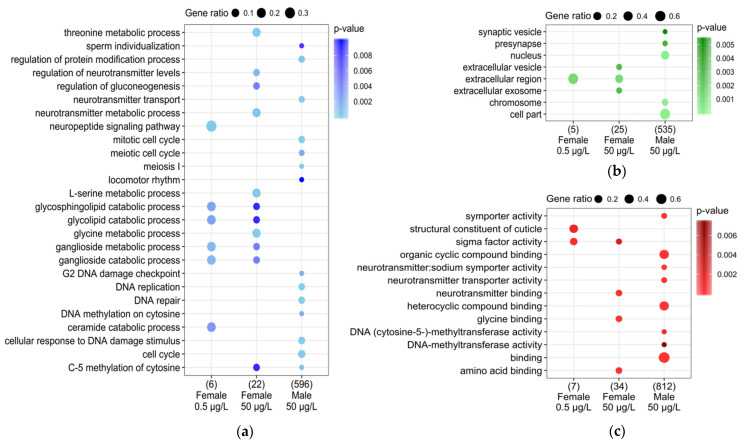
Dotplot of the main GO terms highlighted after the functional enrichment in females and males exposed to 0.5 and/or 50 µg/L of TEB. The functional analysis was performed in three aspects, i.e., (**a**) biological process, (**b**) cellular component, and (**c**) molecular function. GO terms were considered enriched with a Fisher’s exact test *p*-value ≤ 0.01. Dot size, i.e., gene ratio, is the ratio between the number of genes in a given GO term to the total number of mis-regulated genes considered in the GO enrichment analysis (indicated in parentheses on the dot plot).

To gain an overview of the biological functions and pathways of DEGs, we performed a GO and KEGG functional enrichment for both sexes at 50 µg/L and for females at 0.5 µg/L. A sub-selection of the GO terms of interest is represented in Figure 2. We investigated GO term enrichment in three aspects: biological process (BP; Figure 2a), cellular component (CC; Figure 2b), and molecular function (MF; Figure 2c). Table S3 presents the complete list of GO terms (Fisher’s exact test *p*-value ≤ 0.01) and KEGG (FDR ≤ 0.05) pathways that were significantly enriched for both sexes at 50 µg/L and for females at 0.5 µg/L. In males exposed to 50 µg/L, GO terms were mostly related to cell cycle (GO:0007049; *p*-value < 1 × 10^−7^ and ko04110; FDR < 0.05), i.e., the mitotic cell cycle (GO:0000278; *p*-value < 1 × 10^−5^) or DNA replication (GO:0006260; *p*-value < 1 × 10^−9^ and ko03030; FDR < 0.05). This result was confirmed by the analysis for the CC category with terms such as nucleus (GO:0005634; *p*-value < 1 × 10^–9^), chromosome (GO:0005694; *p*-value < 1 × 10^−6^) or cell part (GO:004464; *p*-value < 1 × 10^−6^). Additionally, terms related to reproductive functions, i.e., sperm individualization (GO:0007291; *p*-value < 0.01) or meiosis I (GO:007127; *p*-value < 1 × 10^−3^), and terms corresponding to DNA repair (GO:0006281; *p*-value < 1 × 10^−4^) were highlighted by the enrichment analysis. Furthermore, enrichment occurred in locomotor rhythm (GO:0045475; *p*-value < 0.01) and in terms associated with neurotransmitter transport (GO:006836; *p*-value < 1 × 10^−7^), presynapse (GO:0098793; *p*-value < 0.01) or neurotransmitter:sodium symporter activity (GO:005328; *p*-value < 1 × 10^−6^). Moreover, terms related to DNA methylation such as DNA-methyltransferase activity (GO:0009008; *p*-value < 0.01) and C-5 methylation of cytosine (GO:0090116; *p*-value < 1 × 10^−3^) in males at 50 µg/L were enriched. The latter was shared with females exposed to 50 µg/L (GO:0090116; *p*-value < 0.01). In females, as in males exposed to 50 µg/L, terms related to neurotransmitter metabolic processes (GO:0042133; *p*-value < 1 × 10^−3^) and neurotransmitter binding (GO:0042165; *p*-value < 1 × 10^−3^) were enriched. Similarly, in females exposed to 50 µg/L, glycine, serine, and threonine metabolism (ko00260; FDR < 1 × 10^−3^) and regulation of gluconeogenesis (GO:0006111; *p*-value < 0.01) were enriched. Moreover, terms related to glycolipid, ganglioside, and glycosphingolipid metabolism with p-values < 0.01 and those related to extracellular region (GO:0005579; *p*-value < 0.01) were enriched in females at both concentrations of TEB. Finally, in females exposed to 0.5 µg/L of TEB, the analysis revealed the enrichment of DEGs related to the neuropeptide signaling pathway (GO:0007218; *p*-value < 1 × 10^−3^) and structural constituent of cuticle (GO:0005201; *p*-value < 0.01).

### 3.4. Mis-Regulated Genes of Interest

To complete the gene functional analysis, each gene was systematically reviewed by manually searching known protein names within the Uniprot Knowledgebase database (UniProtKB, https://www.uniprot.org/, accessed on 5 May 2020). We then selected genes of interest based on the mode of action (MoA) of TEB and the effects of these kind of endocrine disruptor model insecticides on moulting processes and putative effects. The selected genes are presented in Table 2.

## 4. Discussion

In the present work, we assessed the effects of a model endocrine disruptor, tebufenozide (TEB), on estuarine copepod *E. affinis*. We performed transcriptomic analysis to provide new insights into the impact of an ecdysone receptor agonist on non-targeted species. Male and female copepods were exposed to concentrations of 0.5 and 50 µg/L to assess sex-specific responses, which is important when assessing the effects of ED.

### 4.1. Sex-Specific Transcriptomic Response

At 0.5 µg/L, no TEB was detected in the medium at T0 and T72h probably because the quantification limit, i.e., 0.4 µg/L, was too close to the nominal concentration. The differential analysis revealed few DEGs in both sexes after 72 h of exposure to TEB for this concentration. Nevertheless, the fact that genes were shared among females at both concentrations suggests that this concentration was worth considering in the present work. Furthermore, as the transcriptomic analysis was performed at a single point in time, it is possible that genes expression was modulated by this concentration of TEB before or after 72 h. 

Differential analysis highlighted a wider number of genes at 50 µg/L, i.e., 116 in females and 1324 in males. This result indicates a sex-specific response to exposure to TEB at the concentration and exposure duration tested in the present work, which is consistent with findings from previous studies on copepods. A higher elevated transcriptomic response in males than in females to endocrine disruptor pesticides, oxidative stress, and cadmium (Cd) has been reported in the copepods *E. affinis*, *Tigriopus californicus*, and *Pseudodiaptomus annandalei,* respectively [33,54,55,56]. Male copepods have been found to be more sensitive to stress for a range of stressors including temperature, salinity, and pollutants such as Cd at higher biological levels [57,58,59]. In the case of metal exposure, this sensitivity was associated with higher concentrations of Cd in male compared to female copepods, suggesting that metal detoxification processes are more efficient in females [60]. This indicates a potential role of a sex-differential metabolization rate of toxic compounds. This higher sensitivity to stressors in male copepods could be attributed to a lower or a latent capacity to cope with stress. This is similar to findings by Boulangé-Lecomte et al [61] of weaker basal expressions of two hsp genes in male *E. affinis* compared to females. The above-mentioned results demonstrate a sex-specific response to exposure to TEB in *E. affinis* and highlight the importance of sex-related studies in ecotoxicogenomics.

### 4.2. Defence Systems and Resistance to Insecticide in Males

In the present work, two glutathione S-transferases (GSTs) and a cytochrome P450 (*cyp6a13*) in males were up-regulated at 50 µg/L, whereas a single esterase (*esterase FE4-like*) was down-regulated. These three enzyme groups have essential roles in insecticide detoxification and thus conferred insecticide resistance [62,63,64]. Additionally, together with direct detoxification processes via the metabolization of compounds or their secondary products, GSTs protect organisms indirectly through their peroxidase activity to cope with oxidative stress produced by insecticides [64]. The reactive oxygen species (ROS) that are potentially produced by exposure to TEB could lead to damage of DNA, and the mis-regulation of genes involved in DNA repair in males at 50 µg/L could be an indication of this damage. Thus, it could be advantageous to assess DNA damages at the cellular level after exposure to TEB. Overall, our results indicate that detoxification processes continued in males after 72 h of exposure to TEB. This was not observed in females; thus, it could be considered support for the hypothesis of a wider latency to cope with stress in males.

Furthermore, the gene coding for *phenoloxidase 2-like* was down-regulated in males exposed to 50 µg/L of TEB, which demonstrates the potential impact of exposure to TEB on the copepod defence response. The phenoloxidase (PO) cascade is essential in innate immune responses in invertebrates, particularly for defence against infection in crustaceans [65,66]. Not only is the PO cascade associated with immunity, but it is also involved in sclerotization of the newly formed cuticle during moulting as well as the repair of damaged cuticles [67]. Thus, according to our results, TEB potentially impacts the cuticle integrity of *E. affinis* through its action as an ecdysone receptor agonist. The potential combined effect of TEB on the defence response and cuticle integrity could lead to greater sensitivity to pathogens and pollutants in copepods. 

### 4.3. Moulting Process and Cuticle Integrity

Three broad complex (Br-c) genes and a single ecdysone-induced protein *74EF-like* (Eip74EF) were mis-regulated in males after exposure to TEB at 50 µg/L. Br-c and Eip74EF are ecdysteroid early responsive genes involved in metamorphosis and moulting [21,68]. As an ecdysone receptor agonist, exposure to TEB is expected to trigger up-regulation of ecdysone early responsive genes that will activate the molecular cascade, resulting in moulting and metamorphosis [36]. In the present study, *Eip74EF* was up-regulated and the three above-mentioned Br-c genes were down-regulated, indicating that, for the latter, the agonist effect ceased to occur. The mis-regulation of these early genes could account for the observed mis-regulation of genes in males at 50 µg/L that were involved later in moulting and metamorphosis such as proteases (i.e., *endochitinase A-like*, *cathepsin L1-like*, *chymotrypsin-like protease CTRL-1*, *chymotrypsinogen A-like*). *Trypsin 1-like* genes were also mis-regulated in both sexes at 50 µg/L. During moulting, the newly formed cuticle is deposited beneath the old one, and the space between them is filled with a moulting fluid whose function is to degrade the old cuticle [69]. In insects, i.e., *Manduca sexta*, different types of proteases, such as trypsin, chymotrypsin, carboxypeptidase, and aminopeptidase, form the composition of the moulting fluid [70]. In the same species, endochitinases, involved in chitin digestion, was found in the integument and moulting fluid [71]. Chitinase and serine and cysteine proteases including trypsins, chymotrypsins, and cathepsins were found to be expressed in premoult in crustaceans [72,73,74,75], during which the old cuticle is degraded and a new one is formed. Thus, the mis-regulation of proteases in copepods in response to exposure to TEB could have an impact on cuticle integrity. This hypothesis could also be extended to female copepods for which genes that code for cuticle proteins were down-regulated at both concentrations of TEB. However, as copepods were fed during the exposure, we cannot exclude the possibility that the mis-regulation of genes that code for proteases was due to their role in food digestion [76,77]. 

In lepidopterans, i.e., TEB-targeted organisms, exposure to TEB seems to induce chitin biosynthesis [34,78]. In the present work, genes that code for proteins involved in gluconeogenesis processes were mis-regulated in both sexes at 50 µg/L (e.g., *fbp-l* or regulation of gluconeogenesis GO:0006111). As glucose enters the chitin biosynthetic pathway, [79] it is probable that exposure to TEB of a non-targeted species, such as the copepod *E. affinis*, induces chitin biosynthesis impairment, which leads to cuticle defects. Furthermore, genes that code for mucins were down-regulated under all conditions (except in males at 0.5 µg/L), and the functional analysis revealed an impact of exposure on threonine/serine metabolism in females at 50 µg/L. Mucins are glycoproteins with serine and/or threonine rich-domains, composing mucous whose functions include cell protection from infection, dehydration, or injuries (physical or chemical), and have a role in the digestive tract [80]. The combined effects of the potential impairment of cuticle integrity and the down-regulation of mucin genes could lead to greater sensitivity to pollutants and/or pathogens, as was proposed earlier. 

Br-c genes have been proposed to play a role in the regulation of methyl farnesoate action [20]. The gene coding for *methyl farnesoate epoxidase-like* was up-regulated in both sexes at 50 µg/L of TEB. In insects, this gene, also known as *cyp15a1*, is responsible for the final conversion of methyl farnesoate (MF) to juvenile hormone III (JHIII) [81]. JHIII is involved in various physiological processes, such as metamorphosis, by preventing ecdysone action [82]. JHIII has not been reported in crustaceans; therefore, MF is considered the functional crustacean JH [83], and *methyl farnesoate epoxidase-like*/*cyp15a1* potentially plays a role in MF degradation [21,84]. Thus, the up-regulation of *methyl farnesoate epoxidase-like* in both sexes at 50 µg/L through the action of TEB could lead to modification of the MF titer and result in metamorphic events with possible detrimental effects on adult copepods.

Overall, exposure to TEB led to the mis-regulation of genes involved in moulting and metamorphosis in adult male and female *E. affinis*. As these two processes are essential for organism development, it would be advantageous to explore the effects of exposure to TEB of insect growth regulators at the naupliar and copepodite stages. Effects on these essential processes could have an important impact on the population level of copepods.

### 4.4. Reproductive Capacity and Vitellogenin Status in Males

Ecdysone early responsive genes are known to be involved in pathways other than moulting and metamorphosis. In addition, Br-c is reported to be involved in reproductive processes such as vitellogenesis [85], oogenesis [86], and embryogenesis [87] in both insects and crustaceans. Recently, *Eip74EF* was proposed to be involved in Drosophila spermatogenesis and male fecundity [88]. In the present study, genes coding for proteins involved in the reproductive process were mis-regulated in males exposed to 50 µg/L of TEB. In particular, we observed an up-regulation of genes coding for neprilysins. Neprolysins, in Drosophila, have a key role in male fertility, particularly *nep-1* [89], which was up-regulated in our study. The *protein white-like* gene was also up-regulated in males at 50 µg/L. In Drosophila, this gene is associated with eye pigmentation and courtship behaviour; mis-localisation or overexpression of this gene has been associated with male–male courtship behaviour [90]. Additionally, genes that code for proteins involved in arthropod gametogenesis, *ATP-dependent RNA helicase vasa-like* and *maternal protein exuperantia-1-like,* were down-regulated in males at 50 µg/L [91,92]. In adult crustaceans, the *vasa* gene is expressed in gonads and is gradually increased during spermatogenesis [93,94]. Furthermore, this gene plays an important role in germ cell specification, development, and maintenance during embryogenesis in arthropods [95]. Thus, mis-regulation of this gene could lead to impairment of the reproductive capacity in adults and issues in germ line development during embryogenesis. This hypothesis is supported by the down-regulation of genes such as *gametogenetin-binding protein-2-like* (*ggnbp2-l*) and *zonadhesin-like* in males at 50 µg/L. A reduction in fertility was observed in male *ggnbp-2-null* mice [96,97]. The zonadhesin protein is a sperm-specific protein localized in the acrosome in mammals and it plays a role in sperm–egg interaction during fertilization [98]. The role of this gene in crustaceans and copepods remains to be elucidated, as no acrosome-like structure has been described in the sperm ultrastructure of copepods [99]. However, it is possible that the genes involved in fertility in mammals could have a similar role in copepods. Considering the TEB MoA as an ecdysone receptor agonist and its effect on ecdysone early genes, these results indicate potential impairment of the reproductive capacity of adult copepods and potential germ line developmental issues during embryogenesis. These effects could have significant impacts on maintenance of the population of these organisms.

In this study, *vitellogenin-like* was down-regulated in males at 50 µg/L. In vertebrates, the induction of vitellogenin (*vtg*) is used as a biomarker of feminisation after exposure to ED; however, researchers focusing on invertebrates have concluded that the use of *vtg* as a biomarker of ED and feminisation is not appropriate [100,101]. *Vgt2* expression was shown to be strongly suppressed in female daphnids after exposure to ecdysteroid 20-hydroxyecdysone (20E). The authors proposed that the induction of *vtg* by certain compounds (e.g., 4-nonyphenol) was a result of the anti-ecdysteroid activity of those compounds [102]. Thus, the observed mis-regulation of *vtg-like* in male copepods may result from TEB mimicking 20E action, more than a marker of feminisation *per se*.

### 4.5. Neuromuscular Pathways in Males and Females

Mis-regulation of genes that code for proteins involved in muscle contraction, such as myosins, actins, or titins, was highlighted in males and females exposed to 50 µg/L. Muscle atrophy and restoration were observed in macro-crustaceans before and after ecdysis, emphasizing the role of ecdysteroids in the muscle growth process [103]. Furthermore, the mis-regulation of muscle-related genes could result from activation of the ecdysone motor program (EMP) involved in shedding of the old cuticle during which ecdysis behaviour occurs, including muscle contraction [104]. As TEB behaves as an ecdysone receptor agonist, it is probable that it affects genes involved in muscle structure and contraction during the moulting process. These alterations in muscle-related genes could have an impact at the individual stage by modifying the swimming behaviour of copepods. This could potentially impair their escape mechanism, food search, and mating. These behaviours additionally depend on proper functioning of neuronal transmission and perception of the environment. GO enrichment analysis in males and females at 50 µg/L of TEB revealed terms linked to neurotransmitter regulation or transport. Furthermore, genes involved in neuronal transmission processes were mostly up-regulated in males. Among them were genes belonging to the innexin family (i.e., *inx2-l* and *shak-B-l*) that code for membrane proteins, which form gap junctions involved in several biological systems, including muscular and nervous systems [105]. Drosophila with mutant *shaking-B* genes, also known as *Passover*, presented defects in the escape behaviour in response to a light-off stimulus. This emphasises the role of this gene in electrical synapse transmission [106,107]. Moreover, there was an up-regulation of genes coding for acetylcholine and glutamate receptors, GABA transporters, and *AChE-l* in males at 50 µg/L. Together, these genes code for proteins with critical roles in regulating neurotransmission and functionality of the neuromuscular system [108]. In this study, the *longitudinals lacking protein-like* (*lola-l*) was one of the down-regulated genes in males. This transcription factor is important for the growth and guidance of the axon and for neuronal projection in a developing olfactory system in Drosophila [109,110]. Furthermore, genes involved in visual perception were mis-regulated in males at 50 µg/L. These genes include (i) *chaoptin-like*, which codes for a photoreceptor cell-adhesion protein and is important in rhabdomere partitioning—the rod equivalent in invertebrates [111,112], (ii) *otx5-A-like*, probably involved in the forebrain and formation of optic vesicle in Xenopus [113] and related to *otd* in Drosophila, which is required for photoreceptor development [114], and (iii) *irreC-rst-like*, which is essential for axonal projection or programmed cell death during compound eye development in Drosophila [115,116]. These results indicate probable impairment of olfactory and visual perception, neuronal transmission, and muscular contraction in adult *E. affinis* after exposure at 50 µg/L for 72 h, albeit the lack of compound eyes in copepods and that the nauplius eye remains the only photoreceptor [117]. It would be beneficial to perform behavioural analysis, e.g., swimming behaviour or response to light stimulus, at the individual level to assess the effects of exposure to TEB. This is because the resulting impairment could lead to detrimental effects at individual and population levels. For instance, the walking activity in the TEB-targeted lepidopteran larvae *Anticarsia gemmatalis* was affected by ingestion of TEB at concentrations of 3.86 mg/mL and 12.16 mg/mL [118].

### 4.6. Epigenetic Modifications

Functional analysis of both sexes at 50 µg/L revealed the presence of GO terms linked to C-5 DNA methylation. Furthermore, genes that code for proteins involved in epigenetic mechanisms, such as DNA methylation/demethylation or histone modifications, were down-regulated in males. Epigenetics was first described in the early 1940s and was later defined as the “study of mitotically and/or meiotically heritable changes in gene functions that cannot be explained by changes in DNA sequence” [119]. Among the heritable changes, DNA methylation remains the most studied. The addition of a methyl group to DNA cytosine residues limits access to proteins that initiate gene expression [120]. Therefore, the mis-regulation of genes that code for proteins involved in C-5 DNA methylation/demethylation that occurs in adult copepods after exposure to TEB can be used to explain mis-regulations of some genes. Furthermore, epigenetic changes could be transferred to next generations, which would lead to impairments of key biological functions. For these reasons, it could be beneficial to assess the global DNA methylation level in adult *E. affinis* exposed to TEB to ascertain epigenetic changes after exposure.

## 5. Conclusions

The aim of the present work was to explore the molecular response of a non-targeted organism to an ecdysone receptor agonist insecticide via a transcriptomic approach. We highlighted the similarities in the mode of action (MoA) of the insecticide on its target species and on the copepod *E. affinis* and the main biological function impacted by exposure to the insecticide. 

**Figure 3:**
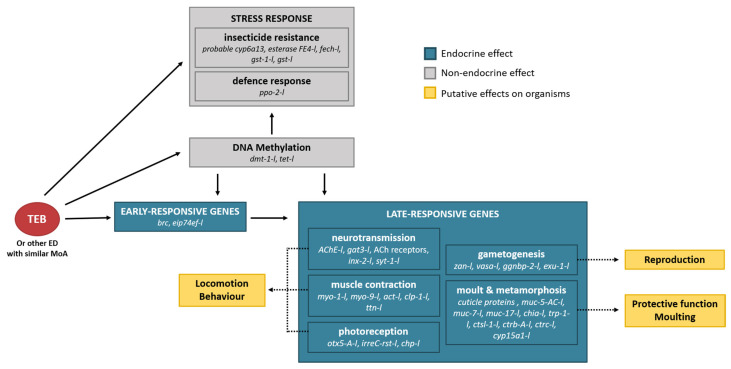
Representation of the putative effects triggered by TEB or other EDs with a similar mode of action on adult male and female *E. affinis*. Grey and blue panels represent the mis-regulated genes and main biological functions observed at the molecular level by the transcriptomic analysis performed in the present work. Yellow panels and dotted arrows represent putative effects triggered by the mis-regulation of those genes at higher biological levels.

The data produced by the transcriptomic analysis allowed the identification of potential biomarkers, including genes specific to the ecdysone receptor agonist MoA as ecdysone early genes, as was proposed in *Gammarus fossarum* [39]. These specific precocious candidates should be complemented by late genes to deploy a battery of biomarkers to accurately identify exposure to ecdysone agonists. Thus, in this study, genes that code for proteins involved in the metabolism of the cuticle and genes involved in the neuromuscular pathways were mis-regulated in both sexes, probably through the action of TEB on early genes. The above-mentioned results are consistent with those of previous studies on the effects of ecdysone receptor agonists on arthropods; effects that lead to the development of an adverse outcome pathway (AOP). Researchers consider early genes as the first key molecular event to result in late key events linked (among others) to impairment of the moulting and neuromuscular system at the tissue/organ level to the final adverse outcome in individuals [104]. Furthermore, down-regulation of *vtg* could be an appropriate biomarker for effects of ecdysone receptor agonists (although not for feminisation), as the *vtg* expression is potentially controlled by ecdysteroids [102]. Together with the above-mentioned potential markers of ecdysone receptor agonists and exposure to insect growth regulators, our results highlight more “classical” indicators of stress, such as genes involved in defence against oxidative stress, DNA repair, insecticide detoxification, and resistance. The present study additionally highlighted genes that code for proteins involved in DNA methylation and histone modification. These genes could be considered biomarkers for epigenetic modification. The present study also allowed the detection of putative physiological responses, which could guide future investigations on higher biological levels: (i) alteration of the cuticle protective function or moulting process, (ii) behavioral impairments, and (iii) reproductive issues. Figure 3 summaries the data obtained by the transcriptomics analysis and presents potential links between gene mis-regulation and potential effects at higher biological levels. Finally, this study reinforces the suitability of the use *E. affinis* as an appropriate ecotoxicological model and reinforces the importance of sex as a factor to be considered in ecotoxicogenomics.

## Figures and Tables

**Table 1:** Concentration of tebufenozide (TEB) in water.

Sample	Time	[TEB] (µg/L)
Control	T0	<QL ^1^
TEB 0.5 µg/L	T0	<QL ^1^
TEB 50 µg/L	T0	13.82
Control	T72h	<QL ^1^
TEB 0.5 µg/L	T72h	<QL ^1^
TEB 50 µg/L	T72h	11.73

^1^ Quantification limit (QL) = 0.4 µg/L.

**Table 2:** Differentially expressed genes (DEGs) of interest after exposure to TEB at 0.5 and 50 µg/L in adult females and males of *E. affinis*. Gene symbols and descriptions were retrieved from the NCBI database and sorted by main biological functions. * and ** respective down- and up- regulated genes with their log2FoldChange. Only significant DEGs are represented; if not significant, cells were -.

	Gene Symbol and Description	♀ 0.5 µg/L	♀ 50 µg/L	♂ 50 µg/L
Moulting and metamorphosis	LOC111704351 broad-complex core protein isoforms 1/2/3/4/5-like	-	-	−1.63 *
	LOC111709681 broad-complex core protein isoforms 1/2/3/4/5-like	-	-	−1.09 *
	LOC111694963 broad-complex core protein isoforms 1/2/3/4/5-like	-	-	−1.49 *
	LOC111704069 ecdysone-induced protein 74EF-like	-	-	1.40 **
	LOC111697606 cuticle protein 7-like	−2.68 *	-	-
	LOC111698563 cuticle protein 7-like	-	−3.02 *	-
	LOC111702984 cuticle protein 16.5-like	−2.18 *	-	-
	LOC111704119 cuticle protein 16.5-like	−1.97 *	-	-
	LOC111708925 cuticle protein 16.5-like	−2.09 *	-	-
	LOC111711095 cuticle protein 16.5-like	-	−1.24 *	-
	LOC111717477 methyl farnesoate epoxidase-like	-	1.53 **	2.97 **
Glycosphingolipids	LOC111699882 ganglioside GM2 activator-like	−1.11 *	-	-
	LOC111702480 ganglioside GM2 activator-like	-	−1.70 *	-
Mucins	LOC111697205 mucin-5AC-like	−1.90 *	−1.88 *	-
	LOC111710342 mucin-5AC-like	-	-	−1.23 *
	LOC111699105 mucin-5AC-like	-	-	−2.95 *
	LOC111716966 mucin-7-like	-	-	−1.26 *
	LOC111700935 mucin-17-like	-	-	−7.06 *
	LOC111713839 mucin-17-like	-	-	−1.02 *
Protease	LOC111697677 cathepsin L1-like	-	-	1.11 **
	LOC111707394 chymotrypsin-like protease CTRL-1	-	-	1.31 **
	LOC111701514 chymotrypsinogen A-like	-	-	1.31 **
	LOC111697179 endochitinase A-like	-	-	−1.26 *
	LOC111716204 trypsin-1-like	-	−1.54 *	-
	LOC111703434 trypsin-1-like	-	-	1.62 **
Muscle	LOC111696032 actin, clone 403-like	-	-	1.51 **
	LOC111717718 actin, muscle-like	-	-	1.09 **
	LOC111697494 calpain clp-1-like	-	-	1.64 **
	LOC111713061 myocyte-specific enhancer factor 2-like	-	-	1.76 **
	LOC111712575 myosin-1-like	-	-	2.96 **
	LOC111695443 myosin-9-like	-	-	−1.44 *
	LOC111711689 myosin heavy chain, muscle-like	-	−2.25 *	−1.92 *
	LOC111711716 myosin heavy chain, muscle-like	-	−3.56 *	−2.86 *
	LOC111715464 myosin heavy chain, muscle-like	-	-	1.61 **
	LOC111708305 myosin regulatory light chain 2-like	-	-	1.08 **
	LOC111703505 titin homolog	-	-	1.34 **
	LOC111697095 titin-like	-	-	1.21 **
Neuro-transmission	LOC111696990 acetylcholinesterase-like	-	-	1.62 **
	LOC111695263 acetylcholine receptor subunit alpha-like	-	-	2.45 **
	LOC111697821 acetylcholine receptor subunit alpha-type acr-16-like	-	-	1.03 **
	LOC111714797 acetylcholine receptor subunit beta-like 2	-	-	1.45 **
	LOC111695314 innexin inx2-like	-	-	−3.75 *
	LOC111702621 innexin inx2-like	-	-	1.60 **
	LOC111718086 innexin shaking-B-like	-	-	1.92 **
	LOC111698781 neurexin-1-like	-	-	1.45 **
	LOC111698578 neurexin-3-like	-	-	1.25 **
	LOC111704195 sodium- and chloride-dependent GABA transporter 3-like	-	-	1.79 **
	LOC111711043 sodium- and chloride-dependent GABA transporter ine-like	-	-	2.61 **
	LOC111708680 synaptotagmin 1-like	-	-	1.33 **
	LOC111697845 synaptotagmin 1-like	-	-	1.75 **
	LOC111703377 synaptotagmin-1-like	-	-	2.86 **
	LOC111715709 glutamate [NMDA] receptor subunit 1-like	-	-	1.41 **
	LOC111700830 glutamate receptor ionotropic, kainate 1-like	-	-	1.28 **
	LOC111715864 glutamate receptor ionotropic, NMDA 2B-like	-	-	2.48 **
	LOC111712426 longitudinals lacking protein-like	-	-	−1.32 *
Visual perception	LOC111695981 chaoptin-like	-	-	−3.02 *
	LOC111698143 homeobox protein otx5-A-like	-	-	−1.46 *
	LOC111708628 irregular chiasm C-roughest protein-like	-	-	1.93 **
	LOC111699563 irregular chiasm C-roughest protein-like	-	-	1.37 **
Defence response-Insecticide resistance	LOC111716773 phenoloxidase 2-like	-	-	−8.32 *
	LOC111705105 probable cytochrome P450 6a13	-	-	1.38 **
	LOC111715882 esterase FE4-like	-	-	−2.46 *
	LOC111698650 ferrochelatase, mitochondrial-like	-	-	−1.56 *
	LOC111698532 glutathione S-transferase 1-like	-	-	1.60 **
	LOC111714366 glutathione S-transferase-like	-	-	1.76 **
DNA methylation	LOC111700762 DNA (cytosine-5)-methyltransferase 1-like	-	-	−1.72 *
	LOC111703921 DNA (cytosine-5)-methyltransferase 1-like	-	-	−1.52 *
	LOC111713817 DNA N6-methyl adenine demethylase-like	-	-	−1.43 *
Reproduction	LOC111695973 neprilysin-1-like	-	-	1.26 **
	LOC111714647 neprilysin-2-like	-	-	1.18 **
	LOC111700675 protein white-like	-	-	1.63 **
	LOC111704362 vitellogenin-like	-	-	−7.78 *
	LOC111702462 ATP-dependent RNA helicase vasa-like	-	-	−3.02 *
	LOC111697048 gametogenetin-binding protein 2-like	-	-	−1.77 *
	LOC111705270 maternal protein exuperantia-1-like	-	-	−2.15 *
	LOC111716440 zonadhesin-like	-	-	−1.58 *
DNA repair	LOC111707470 cell cycle checkpoint protein RAD17-like	-	-	−1.33 *
	LOC111710432 DNA mismatch repair protein Msh6-like	-	-	−1.32 *
	LOC111701439 probable DNA double-strand break repair Rad50 ATPase	-	-	−1.37 *

## Data Availability

Raw and count data are accessible in the GEO repository (https://www.ncbi.nlm.nih.gov/gds accessed on 20 September 2021) under accession number GSE173927.

## Supplementary Materials

The following are available online at https://www.mdpi.com/article/10.3390/genes12101484/s1, Figure S1: *E. affinis* mortality after exposure to tebufenozide (0.5 to 500 µg/L). Each concentration point was performed in triplicate (*n* = 10 per replicate). Mortality was recorded every day along the 96h exposure. Figure S2: Heatmap of the Euclidean distance (a) and PCA (b) from the count matrix for all sequenced samples. Table S1: Number and quality score of raw reads obtained after sequencing. Table S2: List of DEGs in both sexes at 0.5 and 50 µg/L of TEB and list of shared genes between conditions. Table S3: Complete list of GO terms and KEGG pathways significantly enriched for both sexes at 0.5 and 50 µg/L of TEB.
