# Supplementary material for: Susceptibility of the Non-Targeted Crustacean Eurytemora affinis to the Endocrine Disruptor Tebufenozide: A Transcriptomic Approach
Source: Genes (Basel). 2021 Sep 24;12(10):1484. doi: 10.3390/genes12101484 (PMC8536038; doi:10.3390/genes12101484)
Supplement: Supplementary file 1 [file genes-12-01484-s001.zip › genes-1356605-supplementary/Figure S1 S2-CBL.pdf]

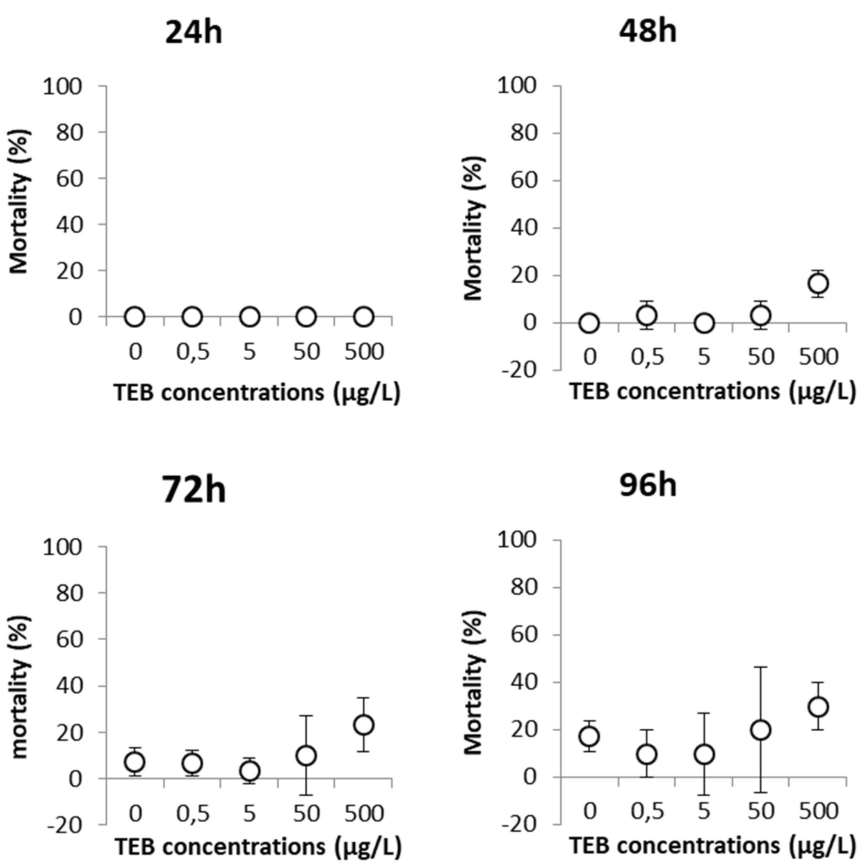

Figure S1. *E. affinis* mortality after exposure to tebufenozide (0.5 to 500 µg/L). Each concentration point was performed in triplicate (n=10 per replicate). Mortality was recorded every day along the 96h exposure.

a

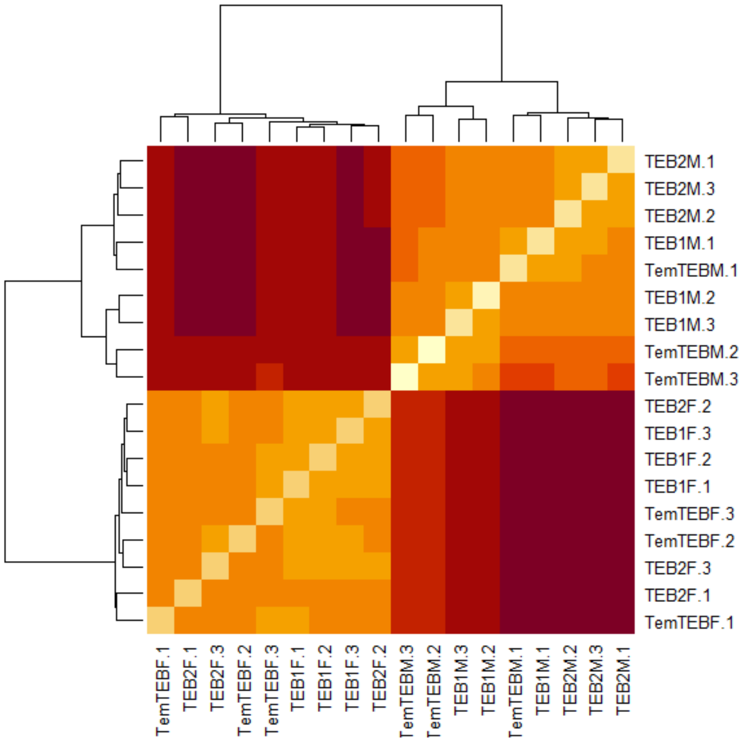

b

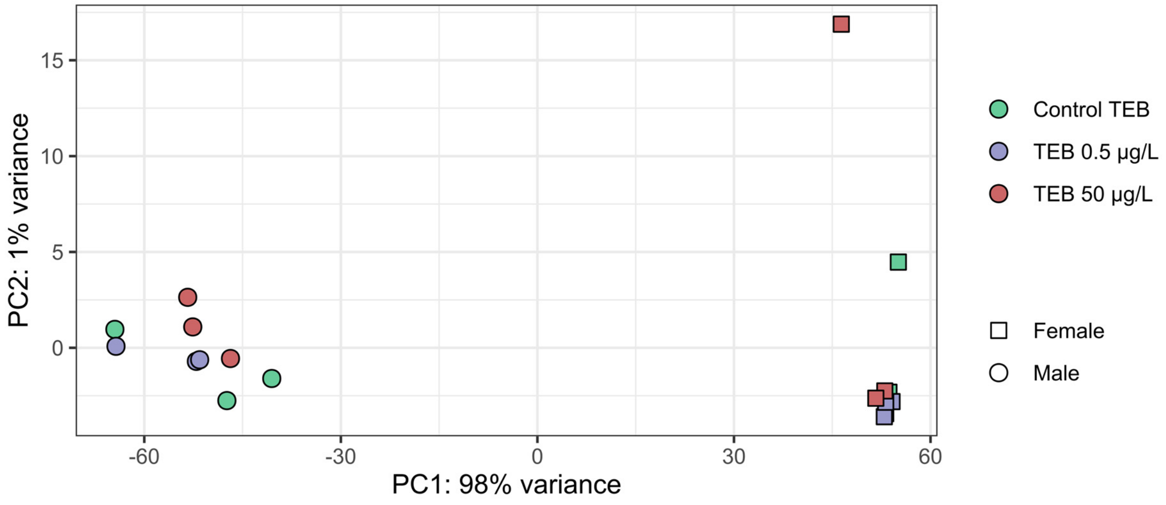

Figure S2. Heatmap of the Euclidean distance (a) and PCA (b) from count matrix for all sequenced samples.
